# MUC1 extracellular domain confers resistance of epithelial cancer cells to anoikis

**DOI:** 10.1038/cddis.2014.421

**Published:** 2014-10-02

**Authors:** Q Zhao, T Piyush, C Chen, M A Hollingsworth, J Hilkens, J M Rhodes, L-G Yu

**Affiliations:** 1 Department of Gastroenterology, Institute of Translational Medicine, University of Liverpool, Liverpool, UK; 2 Department of Biochemistry and Molecular Biology, The Eppley Institute for Research in Cancer and Allied Diseases, University of Nebraska Medical Centre, Omaha, NE, USA; 3 Division of Molecular Genetics, the Netherlands Cancer Institute, Amsterdam, The Netherlands

## Abstract

Anoikis, a special apoptotic process occurring in response to loss of cell adhesion to the extracellular matrix, is a fundamental surveillance process for maintaining tissue homeostasis. Resistance to anoikis characterises cancer cells and is a pre-requisite for metastasis. This study shows that overexpression of the transmembrane mucin protein MUC1 prevents initiation of anoikis in epithelial cancer cells in response to loss of adhesion. We show that this effect is largely attributed to the elongated and heavily glycosylated extracellular domain of MUC1 that protrudes high above the cell membrane and hence prevents activation of the cell surface anoikis-initiating molecules such as integrins and death receptors by providing them a mechanically ‘homing' microenvironment. As overexpression of MUC1 is a common feature of epithelial cancers and as resistance to anoikis is a hallmark of both oncogenic epithelial–mesenchymal transition and metastasis, MUC1-mediated cell resistance to anoikis may represent one of the fundamental regulatory mechanisms in tumourigenesis and metastasis.

Anoikis, the apoptotic process that occurs in cells that have lost adhesion to the extracellular matrix (ECM),^1,2^ is a fundamental process for maintaining tissue homeostasis. It removes displaced epithelial/endothelial cells and thus prevents them from seeding to inappropriate sites. Resistance to anoikis contributes prominently to tumourigenesis and, in particular, to metastasis by allowing survival of cancer cells that have invaded into the blood or lymphatic circulation and thus facilitating their metastatic spread to remote sites.^3^

Initiation of anoikis starts from the cell surface through activation of the cell surface anoikis-initiating molecules, for example, integrins, cadherins and death receptors, in response to loss of cell adhesion. Loss of the integrin-mediated cell basement matrix contact,^4^ loss of the E-cadherin-mediated cell–cell contact^5,6^ or ligation of the cell surface death receptors with their ligands^4,7^ all induce conformational changes or oligomerization of these cell surface anoikis-initiating molecules. This triggers a series of events leading to activation of either the caspase-8-mediated extrinsic apoptotic signalling pathway or the mitochondrion-mediated intrinsic apoptotic signalling pathway.

MUC1 is a large transmembrane mucin protein that is expressed exclusively on the apical side of normal epithelial and some other cell types. MUC1 consists of a large extracellular domain, a transmembrane region and a short cytoplasmic tail. The MUC1 extracellular domain contains a variable number of tandem repeats that are heavily glycosylated (up to 50% of the MUC1 molecular weight) with complex *O*-linked mucin-type glycans^8^ and flanked by a unique N-terminal domain and an SEA domain. In the SEA domain, autocleavage takes place resulting in a heterodimer but both moieties remain firmly attached. The cytoplasmic tail of MUC1 contains 72 amino acids and harbours several phosphorylation sites and is able to interact with various growth factor receptors and intracellular signalling proteins.^9, 10, 11^

MUC1 is overexpressed up to at least 10-fold in epithelial cancers^12^ and overexpression of MUC1 is closely associated with high metastatic potential and poor prognosis in many cancer types.^13^ In epithelial cancer cells, MUC1 loses its apical membrane polarization and becomes expressed over the entire cell surface.^14,15^ In epithelial cancer cells, MUC1 also shows reduced expression of complex *O*-glycans and increased expression of short oncofetal oligosaccharides such as GalNAc-*α* (Tn antigen), sialylated GalNAc-*α* (sialyl-Tn antigen) and Gal*β*1,3GalNAc-*α* (Thomsen–Friedenreich, TF antigen).^16^ Immunological targeting of cancer-associated MUC1 has been under intensive investigation as a strategy for cancer treatment.^17,18^ Our recent studies have shown that interaction of TF antigen on cancer-associated MUC1 with the galactoside-binding galectins promotes metastasis by enhancing tumour cell heterotypic adhesion to the vascular endothelium and also by increasing tumour cell homotypic aggregation for the potential formation of tumour emboli.^19–21^

In this report, we describe a new role of MUC1 in anoikis. We show that overexpression of MUC1 in epithelial cells prevents initiation of anoikis in response to loss of cell adhesion, an effect that is found to be attributed substantially to the MUC1 extracellular domain.

## Results

### Overexpression of MUC1 is associated with increased cell resistance to anoikis

**Figure 1:**
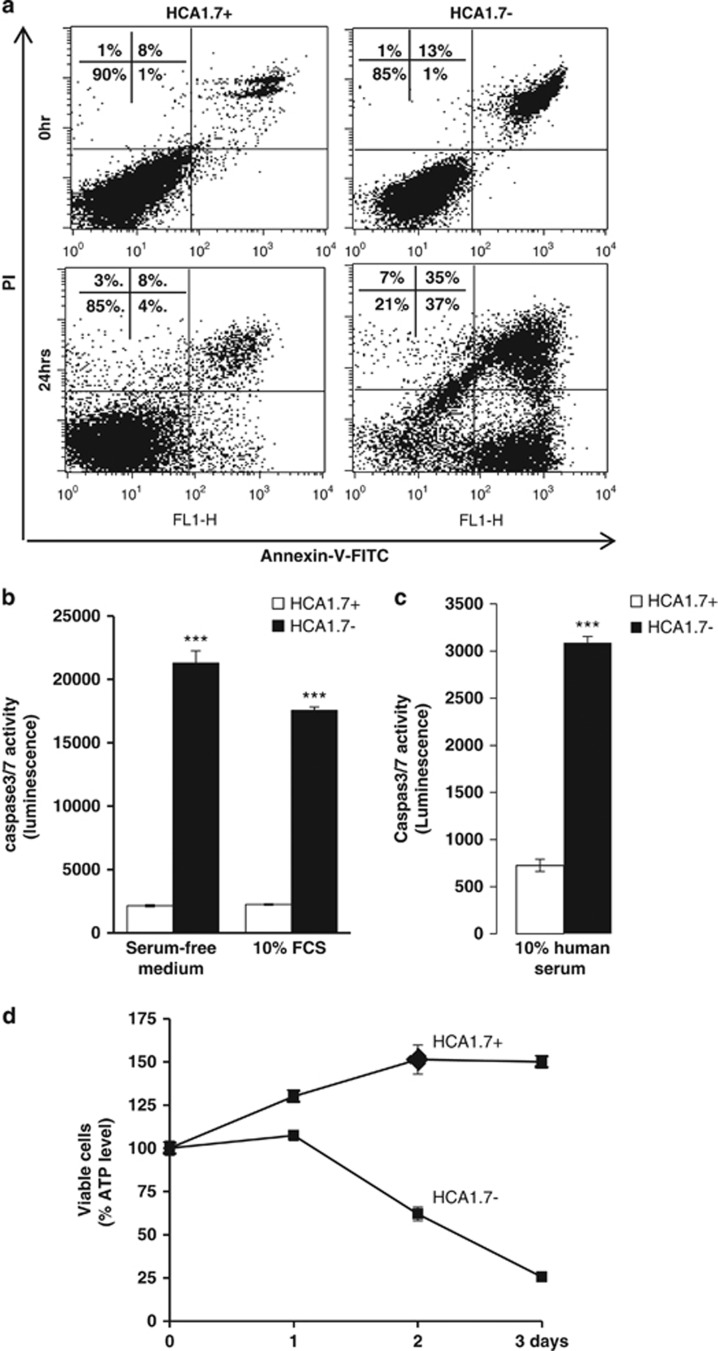
MUC1 transfection in human breast epithelial HBL-100 cells inhibits anoikis and increases cell survival. (**a**) Representative flow cytometry plots showing Annexin-V cell surface binding of the MUC1-positive (HCA.17+) and -negative (HCA1.7−) transfectants, released by NECDS and cultured for 0 and 24 h in suspension. Earlier apoptotic (Annexin-V-positive and PI-negative) cells shown at the bottom right and late apoptotic (Annexin-V-positive and PI-positive) cells shown at the top right in each of the correlation plots. (**b**, **c**) Assessment of caspase-3/-7 activity of HCA1.7+/− cells in cell response to 24 h culture in suspension in serum-free medium, 10% FCS (**b**) or 10% human serum (**c**). The data are presented as mean±S.E.M. of triplicate determinations from two independent experiments. (**d**) MUC1 expression increases cell viability in response to cell culture under suspension. The data are presented as mean±S.D. of triplicate determinations. ***P<0.001

**Figure 2:**
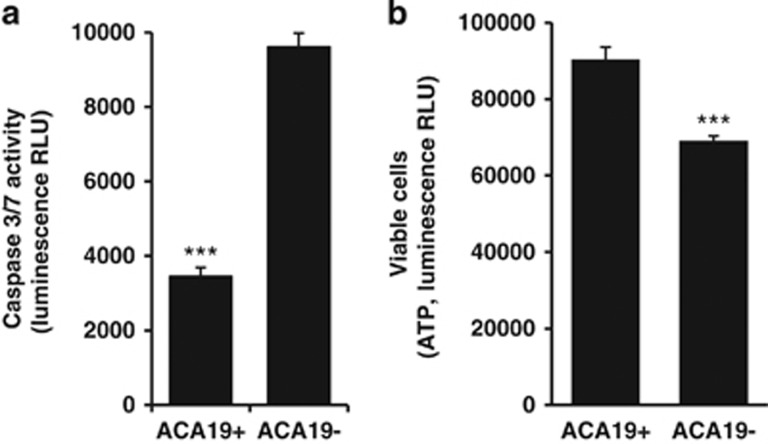
MUC1 expression in human melanoma cells prevents anoikis and increases cell survival. MUC1-positive transfectants (ACA19+) show significantly less anoikis (**a**) and higher survival rate (**b**) than the MUC1-negative revertants (ACA19−) in cell response to 24 h culture under suspension when assessed by caspase-3/-7 activity. The data are presented as mean±S.E.M. of triplicate determinations of two independent experiments. ***P<0.001

MUC1-positive transfectants of human breast HBL-100 epithelial cells (HCA1.7+) showed marked resistance to anoikis in comparison to the MUC1-negative revertants (HCA1.7−) when released by ENCDS and cultured in suspension. After 24 h culture in suspension, 6.1-fold more HCA1.7− cells became apoptotic compared with HCA1.7+ cells when assessed by Annexin-V cell surface binding (Figure 1a). When caspase-3/-7 activity was assessed, HCA1.7+ also showed substantially less casapse-3/-7 activity than HCA1.7− cells after culture of the cells either in serum-free medium, in 10% FCS (Figure 1b) or in human serum (Figure 1c). Consistent with their increased ability to resist anoikis, HCA1.7+ cells also showed substantially higher survival rates than HCA1.7− cells when cultured in suspension (Figure 1d). Similar results were also observed with MUC1-transfected human melanoma cells (Figure 2). After 24 h culture in suspension, the MUC1-positive ACA19+ cells showed much lower caspase-3/-7 activity (Figure 2a) and higher viability (Figure 2b) than the MUC1-negative ACA19− cells.

### Trypsin- and NECDS-released MUC1-positive and -negative cells show different responses to anoikis initiation

To gain insight into the molecular mechanism of the MUC1-mediated cell resistance to anoikis, we investigated the impact of the use of non-enzymatic cell dissociation solution (NECDS) and trypsin for cell release on anoikis initiation of MUC1-positive and -negative cells. NECDS releases the cells from culture plates but keeps the cell membrane proteins intact, whereas trypsin releases the cells by proteolytic cleavage of lysine and arginine residues of extracellular domains of cell membrane proteins.

**Figure 3:**
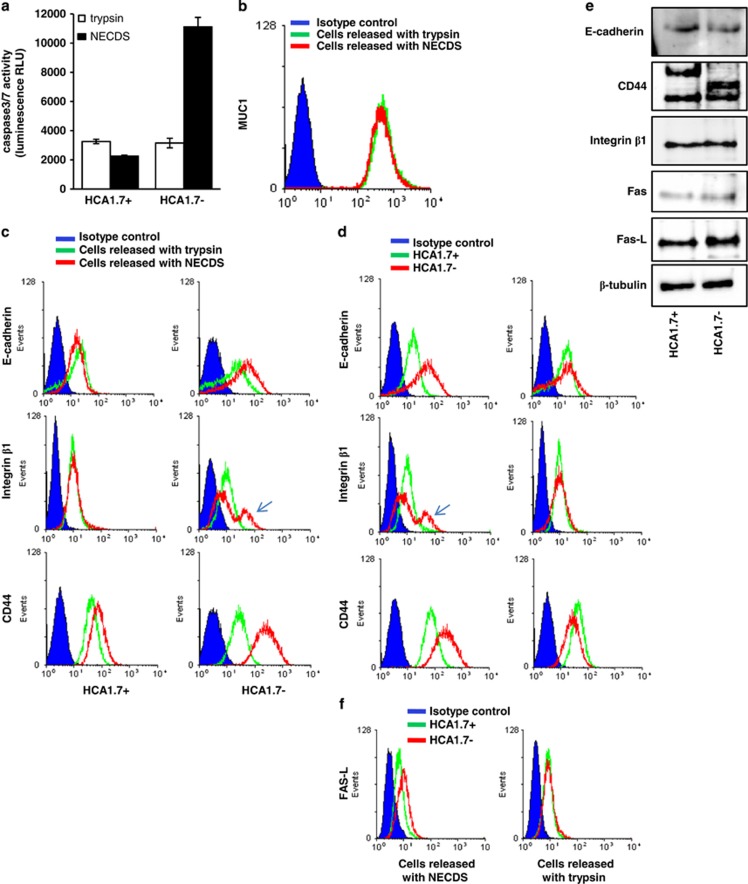
Different effects of cell release by trypsin and NECDS on subsequent initiation of anoikis and on antibody accessibility to the cell surface anoikis-initiating molecules in MUC1-positive and -negative cells. (**a**) HCA1.7+/− cells were released by NECDS or trypsin and cultured in suspension for 24 h before the cell-associated caspase-3/-7 activities were assessed. The data are presented as mean±S.E.M. of triplicate determinations of two independent experiments. (**b**–**d**) Representative flow cytometry plots show antibody access to cell surface MUC1 (**b**), E-cadherin, Integrin*β*1, CD44 (**c**, **d**) and recombinant Fas-L access to cell surface Fas (**f**) in HCA1.7+/− cells released by trypsin or NECDS. Note, an additional integrin*β*1 peak (arrowed) is seen in HCA1.7− cells released by NECDS in comparison to those released by trypsin. (**e**) Immunoblotting of cell lysates shows total cellular expression of CD44, E-cadherin, integrin*β*1, Fas, Fas-L and tubulin in HCA1.7+/− cells

We found that detachment of the MUC1-positive HCA1.7+ cells with either NECDS or trypsin had no significant effect on resistance of the cells to anoikis in response to subsequent culture in suspension (Figure 3a). However, detachment of the MUC1-negative HCA1.7− cells with trypsin completely abolished the sensitivity of these cells to anoikis initiation in response to suspension culture, whereas these MUC1-negative cells remained fully sensitive to anoikis when detached by NECDS. Detachment of the MUC1-positive HCA1.7+ cells with either trypsin or NECDS did not affect anti-MUC1 antibody accessibility to MUC1 (Figure 3b). This indicates that trypsin is unable to cleave the MUC1 extracellular domain, likely due to the large and heavy glycosylated extracellular domain that protrudes above the cell surface and prevents the access of trypsin to the protein backbone of MUC1. Accessibility of antibodies to cell surface antigens like E-cadherin and integrin*β*1 on MUC1-positive HCA1.7+ cells released by trypsin or NECDS shows little difference, with the exception of CD44, which shows 23% less binding to trypsin-released than NECDS-released cells (Figure 3c). On the other hand, the MUC1-negative HCA1.7− cells released by trypsin showed 29, 23 and 85%, respectively, lower antibody accessibility to cell surface E-cadherin, Integrin*β*1 and CD44 than those released by NECDS (Figure 3c). In the meantime, when the HCA1.7+ and HCA1.7− cells were compared, antibody accessibility to the cell surface domains of E-cadherin, integrin*β*1 and CD44 was substantially higher (53, 20 and 83%, respectively) in the MUC1-negative HCA1.7− cells than in the MUC1-positive HCA1.7+ when they were released by NECDS (Figure 3d, left panel). However, when the cells were detached by trypsin, little differences of antibody accessibility were observed between these two cell types (Figure 3d, right panel).

As western blot analysis of the denatured cell lysate showed no difference in protein expression of these cell adhesion molecules between HCA1.7+ and HCA1.7− cells (Figure 3e; note an extra, slightly higher molecular weight CD44 band in HCA1.7− than in HCA1.7+ cells), the restricted access of trypsin to cell surface proteins on MUC1-positive cells likely explains the differences of the effect of trypsin on antibody accessibility to these cell surface molecules on both cell types. This is supported by the discovery that recombinant Fas-L showed 33.2% more binding to HCA1.7− cells than to HCA1.7+ cells when they were released by NECDS, but such difference totally disappeared when the cells were released by trypsin (Figure 3f). Together, these findings indicate that MUC1 expression maintains the integrity of cell surface proteins and prevents activation of cell surface anoikis-initiating molecule(s) during the process of cell loss of adhesion. In support of this, an additional cell surface integrin*β*1 population occurred in the MUC1-negative HCA1.7− cells released by NECDS, which subsequently underwent anoikis, but not in those released by trypsin, which did not undergo anoikis (Figure 3a). Also, no difference of the cell surface integrin*β*1 expression was seen in the MUC1-positive HCA1.7+ cells released by NECDS or trypsin (Figures 3c and d), which did not undergo anoikis. Thus, this additional integrin *β*1 population in HCA1.7− cells released by NECDS might represent the activated ‘open' form (see Discussion below) of this molecule that is involved in anoikis activation.

### MUC1 expression inhibits induction of anoikis induced by exogenous Fas-L

**Figure 4:**
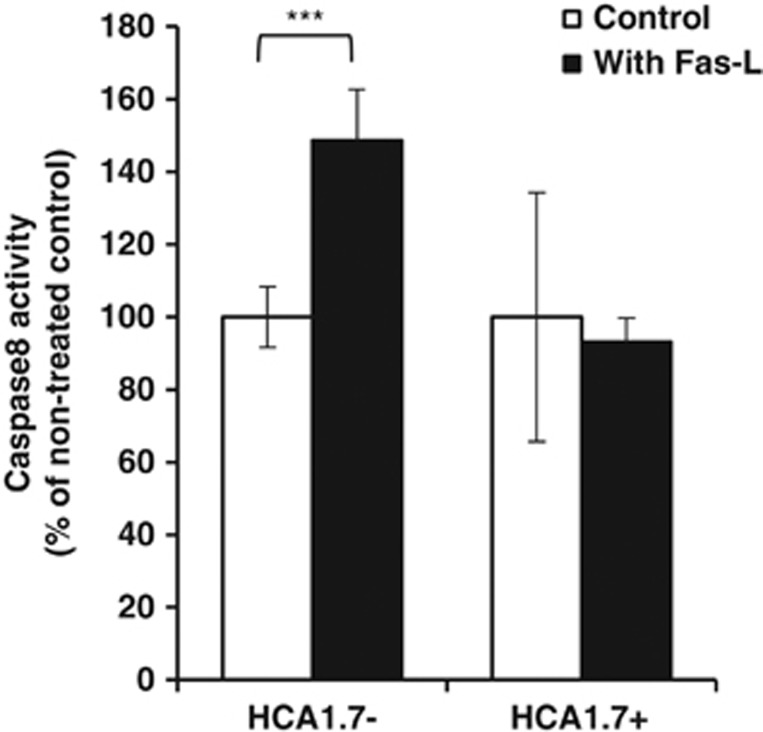
Differential effect of exogenous addition of Fas-L on anoikis of MUC1-positive and -negative cells. HCA1.7+/− cells were treated with 100 ng/ml recombinant Fas-L under suspension for 0 and 2 h followed by assessment of cellular casapse-3/-7 activity. The data are presented as mean ±S.E.M. of triplicate determinations of two independent experiments. ***P<0.001

To further substantiate the association between MUC1 expression and anoikis resistance, we compared the ability of exogenous Fas-L to induce initiation of anoikis of MUC1-positive and -negative cells in suspension. Fas-L binds to cell surface Fas, resulting in activation of caspase-8 and the initiation of extrinsic apoptotic signalling in anoikis. It was found that the presence of 100 ng/ml recombinant Fas-L induced 48% increase in caspase-8 activation of MUC1-negative HCA1.7− cells but had no effect on caspase-8 activation of the MUC1-positive HCA1.7+ cells (Figure 4). This supports the hypothesis that expression of MUC1 on the cell surface prevents activation of the cell surface anoikis-initiating molecules.

### Effects of the MUC1 extracellular and intracellular domains on anoikis

**Figure 5:**
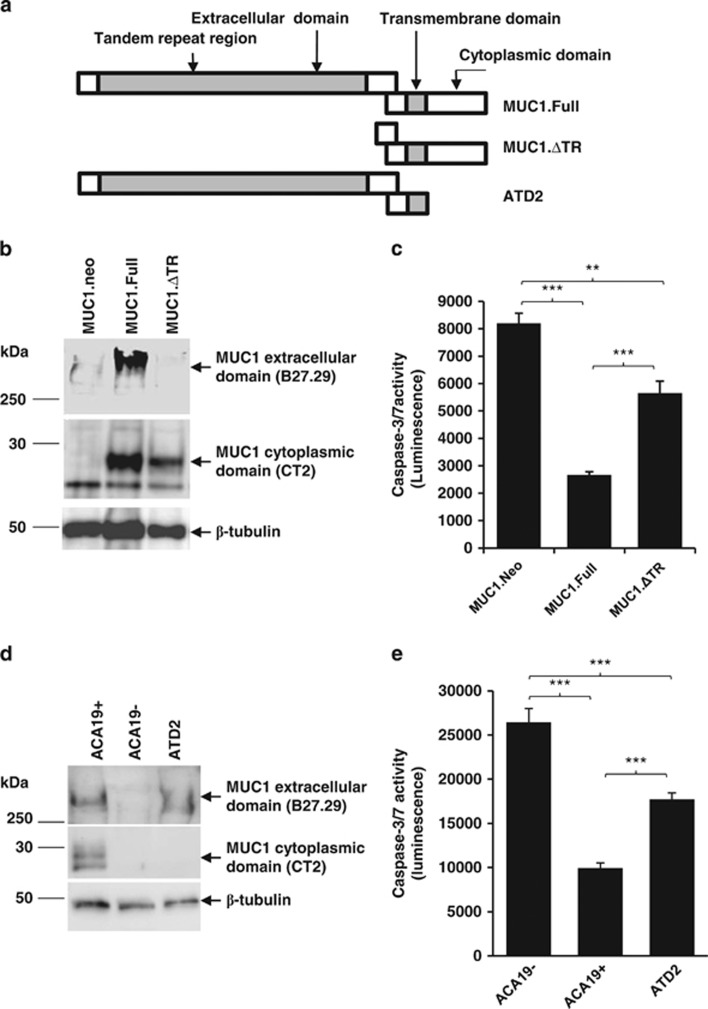
Depletion of MUC1 extracellular tandem repeat domain or cytoplasmic domain reduces MUC1-mediated cell resistance to anoikis. (**a**) Schematic diagram of MUC1 transfectants. (**b**) MUC1 immunoblotting confirms transfection of MUC1 full length and extracellular tandem repeat domain-depleted mutants. HCT116 cells transfected with control vecter (MUC1.neo), full-length MUC1 (MUC1.Full) or MUC1 tandem repeat domain depletion (MUC1.ΔTR) were separated by SDS-PAGE and immunoblotted with B27.29 (against the extracellular tandem repeat domain of MUC1), CT2 (against the cytoplasmic domain) or an anti-tubulin antibody. MUC1.Full cells show expression of MUC1, both extracellular and intracellular, domains and MUC1.ΔTR cells show expression of only the intracellular MUC1 domain. (**c**) Depletion of MUC1 tandem repeat domain reduces MUC1-mediated cell resistance to anoikis when cellular caspase-3/-7 activity was assessed. (**d**) MUC1 transfectants of A375 cells with control vector (ACA19−), full-length MUC1 (ACA19+) or full-length MUC1 without the cytoplasmic domain (ATD2) were immunoblotted with B27.29, CT2 or tubulin antibody. ACA19+ cells show expression of MUC1 extracellular and intracellular domains and ATD2 cells show expression of MUC1 extracellular but not cytoplasmic domain. (**e**) The absence of MUC1 cytoplasmic domain reduces full MUC1-mediated cell resistance to anoikis when cellular caspase-3/-7 activity was assessed. The data are presented as mean ±S.D. of triplicate determinations of two (**c**) or three (**e**) experiments. **P<0.01, ***P<0.001

To test the role of the extracellular domain of MUC1 in resistance to anoikis, MUC1-negative HCT116 cells were transfected with a construct containing full-length MUC1 (MUC1.Full) and a construct containing truncated MUC1 cDNA devoid of the tandem repeat domain (MUC1ΔTR; Figure 5a). Immunoblotting experiments confirmed expression of the correct MUC1 mutants and provide a measure for the expression levels in these transfectants (Figure 5b). Suspension culture of the cells transfected with MUC1.Full resulted in 68% reduction of anoikis in comparison with transfection of the cells with the control vector (Figure 5c), whereas transfection of the cells with MUC1 without its tandem repeat domain gives significantly less resistance to anoikis. This provides strong support for a substantial role of the extracellular domain in MUC1-mediated resistance to anoikis. Interestingly, we observed that transfection of the cells with MUC1 without its extracellular domain also produced a smaller (31%) but significant reduction of cell anoikis in comparison with the control vector transfectants. This indicates that the MUC1 cytoplasmic domain (CT) may also contribute to the MUC1-mediated anoikis resistance.

To test this possibility, we compared A375 cells transfected with full-length MUC1 and a truncated MUC1 construct without cytoplasmic domain for sensitivity to anoikis. A375 cells expressing full-length MUC1 (ACA19+ cells) were highly resistant to anoikis in comparison with the MUC1-negative transfectants (ACA19−) when cultured in suspension (Figures 5d and e), similar as demonstrated earlier (Figures 2a and b). MUC1 transfectants without the MUC1 cytoplasmic domain (ATD2) showed significantly higher anoikis (~50%) than the ACA19+ cells expressing full-length MUC1 but significant less than the MUC1-negative ACA19− cells. This supports an independent anoikis resistance mechanism mediated by the cytoplasmic domain of MUC1 in addition to the anoikis resistance mediated by the extracellular domain of MUC1.

### Effect of MUC1 on expression of apoptosis-related signalling proteins in cell response to culture in suspension

**Figure 6:**
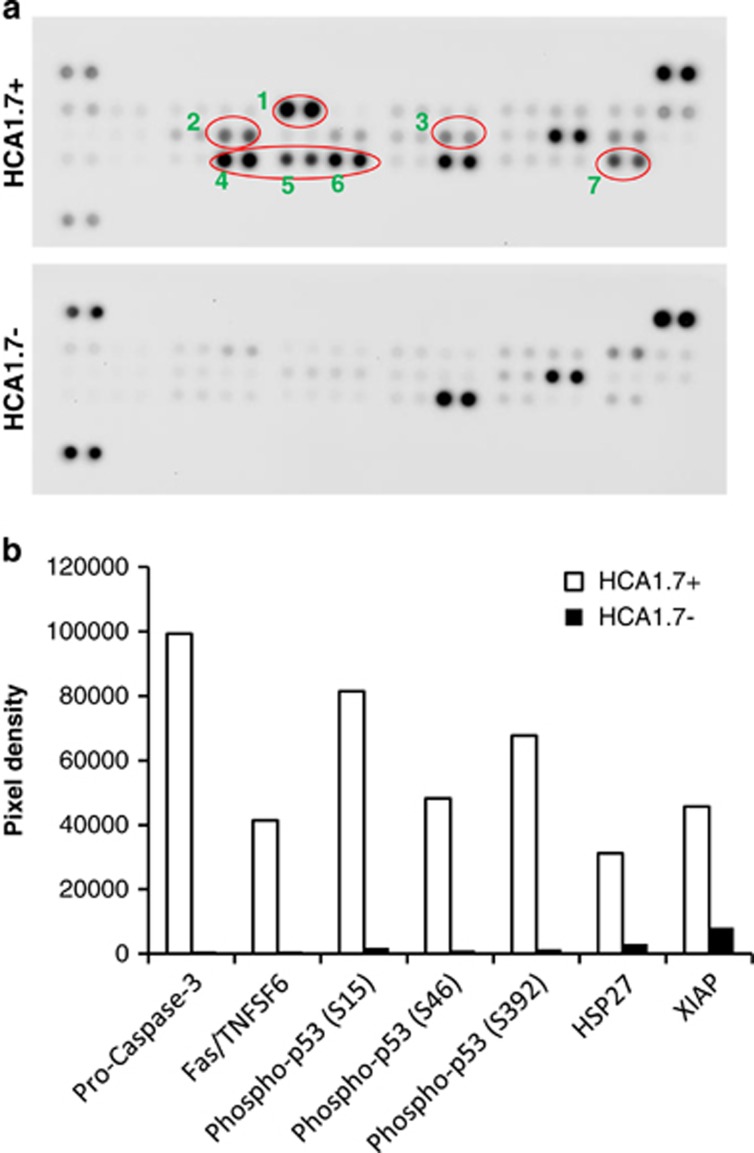
Analysis of the expression of 35 apoptosis-related proteins in HCA1.7+/− cells in response to suspension culture by protein array. HCA1.7+/− cells released by NECDS and cultured at 1 × 10^5^ cells/ml in serum-free DMEM for 24 h in poly-HEMA-coated plates at 37 °C. The cells were collected and lysed and applied to the Human Apoptosis Array. The array contains 35 apoptosis-related proteins. In response to 24 h culture in suspension, five proteins show substantial increased expression (or phosphorylation) in HCA1.7+ cells in comparison with HCA1.7− cells (**a**: 1, pro-caspase-3; 2, Fas; 3, Hsp27; 4, phosphor-p53(S15); 5, phosphor-p53(S46); 6, phosphor-p53(S392); 7, XIAP; **b**: densitometry quantification of the expression (or phosphorylation) of the five proteins in **a**)

To gain further insight into the regulation of MUC1-mediated cell resistance to anoikis, we compare the expressions of 35 apoptosis-related signalling proteins in the MUC1-positive (HCA1.7+) and -negative (HCA1.7−) cells in cell response to suspension culture. We found that among the 35 apoptosis-related proteins, 4 show a substantial increase in expression in the MUC1-positive transfectants in comparison with the MUC1-negative transfectants in response to 24 h culture in suspension (Figures 6a and b). These proteins are XIAP (inhibitor of apoptosis protein, 6-fold increase), Fas (51-fold), HSP27 (10-fold) and pro-caspase-3 (115-fold). In addition, we found an increase in the phosphorylation of several p53 serine residues (S15, 44-fold; S46, 46-fold; S392 46-fold). As the MUC1 cytoplasmic domain is known to interact directly with p53 in apoptosis regulation,^22^ the observed change in p53 phosphorylation in MUC1-positive cells is in keeping with an impact of the MUC1 cytoplasmic domain in MUC1-mediated anoikis resistance shown in Figure 5e.

## Discussion

This study shows that expression of the transmembrane mucin protein MUC1 confers resistance of epithelial cells to anoikis initiation in response to loss of cell adhesion. This effect is found to be substantially attributed to the extracellular domain of MUC1, which prevents the normal activation of cell surface anoikis-initiating molecules in response to loss of cell adhesion.

Anoikis is an important surveillance process for preventing cells from seeding to inappropriate sites. Anoikis is accomplished by a diversity of proteins in the ECM and a range of the cell surface anoikis-initiating molecules, which initiate mitogenic signals in the normal cellular environment or apoptotic signals in the context of abnormal cell contact.^7^ Key cell surface anoikis-initiating molecules include integrins, E-cadherin and death receptors.

Integrins are a family of heterodimers that mediate cation-dependent cell adhesion in a wide range of biological contexts. The integrin family is comprised of 18*α* and 8*β* subunits, which on ligation give rise to 24 different types of integrins. Every cell has integrins that are specific to their ligands in the ECM. Integrins sense mechanical forces arising from contacts with the ECM and converts them into intracellular signals. Integrins have two alternative conformations, a closed, low-affinity ligand-binding conformation and an open, high-affinity ligand-binding conformation.^23, 24, 25^ The open conformation has >9000-fold higher affinity to its ligands than closed conformation. In response to external signals, including loss of the cell surface integrin engagement with ECM, integrin undergoes rapid transition from the closed to the open conformation that triggers inactivation of the pro-survival signalling pathways such as those mediated by FAK, ERK and PI3K, leading to activation of the mitochondrion-mediated apoptotic signalling and execution of apoptosis.^7^ The conformational changes of integrins at the extracellular side can also mediate inside–out signalling.^26^

It is shown here that detachment of the MUC1-negative cells by NECDS, which leads to anoikis (Figure 3a), is associated with the appearance of an additional cell surface integrin*β*1 population (Figures 3c and d). Cells released by trypsin showed the expression of only one integrin*β*1population and were resistant to anoikis (Figures 3a, c and d). Thus, the two different integrin*β*1 populations in the MUC1-negative cells released by NECDS might represent the open (active) and closed (inactive) integrin conformations of integrin*β*1 as a result of cell detachment and activation of integrin*β*1. This is in keeping with the observation that detachment of the MUC1-positive cells by NECDS, to which the cells do not undergo subsequent anoikis (Figure 3a), showed the presence of one integrin*β*1 population (Figure 3c). The presence of MUC1 therefore prevented the activation of integrin*β*1 during cell detachment by NECSD and subsequent anoikis response.

Ligation of extracellular death receptor ligands to their transmembrane death receptors is also known to have an important role in initiation of anoikis in response to cell loss of adhesion. Binding of death receptor ligands (for example, Fas-L) to the extracellular domain of death receptors (for example, Fas) induces death receptor oligomerization that allows the recruitment to the death receptor cytoplasmic domain of several cytoplasmic proteins (for example, Fas-associated protein with death domain (FADD)) and pro-caspase-8, leading to caspase-8 activation and eventual activation of executioner caspases-3/-7.^4^ We found here that exogenous addition of Fas-L induced caspase-8 activation of the MUC1-negative cells but not the MUC1-positive cells in suspension culture (Figure 4), although these cells express similar levels of Fas (Figure 3e). This indicates that the expression of MUC1 not only prevents integrin-mediated anoikis initiation by preventing integrin activation, but also inhibits death receptor-mediated anoikis initiation by preventing ligation of the cell surface death receptors with their ligands in cell response to loss of adhesion.

**Figure 7:**
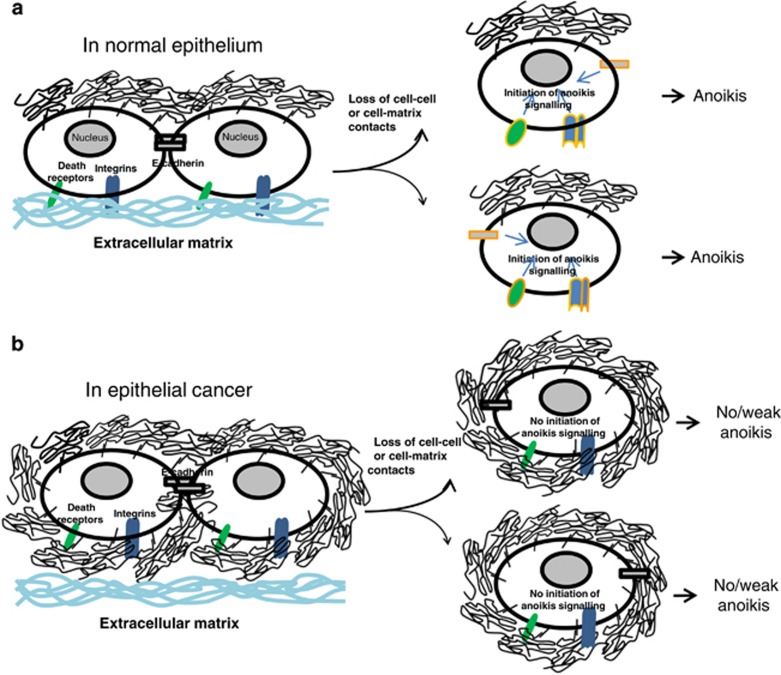
Proposed action of MUC1 extracellular domain in MUC1-mediated cell resistance to anoikis. (**a**) In normal epithelia, MUC1 polarizes at the apical side and has no influence on activation of the cell surface anoikis-initiating molecules during loss of cell-matrix contacts. (**b**) In epithelial cancer, MUC1 is overexpressed over the entire cell surface and thus able to interact with the cell surface anoikis-initiating molecules around the circumference of the cell, preventing their activation during loss of cell-matrix contacts by providing them a mechanically ‘homing' microenvironment

Such a relatively broad influence of MUC1 expression on activation of the cell surface anoikis-initiating molecules is likely explained by its massive size, as the heavily glycosylated MUC1 molecule protrudes up to 10 times higher above the cell surface than other typical cell surface molecules that do not reach farther than 30 nm. The extracellular domain of MUC1 may thus provide the cell surface anoikis-initiating molecules with a ‘homing' microenvironment even after the cells are detached, preventing conformational changes of these molecules and thus inhibiting anoikis (Figure 7).

It was found in this study that depletion of the MUC1 extracellular domain abolishes ~61% of the MUC1-mediated cell resistance to anoikis (Figure 5c), suggesting a predominant role of the MUC1 extracellular domain in MUC1-mediated cell resistance to anoikis. We also found that MUC1 transfection without the extracellular domain still causes anoikis inhibition of the cells, albeit much less efficiently. This indicates that the MUC1 cytoplasmic domain also makes significant contribution to the MUC1-mediated cell resistance to anoikis through different mechanisms. Both intrinsic and extrinsic apoptotic pathways are known to have an important role in anoikis and many of the intrinsic apoptotic signalling proteins (for example, Bcl and p53 family members) are involved in regulation of anoikis process.^27^ Several earlier studies have reported a role of the MUC1 cytoplasmic domain in regulation of apoptosis in cells growing under (anchored) adhesion conditions through interaction with a number of intracellular signalling proteins.^9^ For example, interaction of the MUC1 cytoplasmic tail with mitochondrial membrane, p53 or *β*-catenin prevents mitochondrion-mediated apoptosis in cell response to DNA damage.^10^ Interaction of the MUC1 cytoplasmic tail with FADD blocks caspase-8 recruitment to the death-inducing signalling complex in response to TNF*α*-induced apoptosis.^28^ MUC1 transfection in rat 3Y1 fibroblast cells has been shown to increase the levels of phospho-Akt and phospho-Bad and increase the expression of anti-apoptotic Bcl-x(L) protein. This is accompanied by attenuation of the loss of mitochondrial transmembrane potential, mitochondrial cytochrome c release and activation of caspase-9.^29^ As many intracellular apoptotic signalling events that occur in response to stress also occur in anoikis,^30^ interactions of the MUC1 cytoplasmic domain with these intracellular signalling proteins seen in cell response to stress likely contribute to the lesser inhibition of anoikis that is shown to be associated with the MUC1 cytoplasmic domain (Figures 5c and e).

Substantial increases in the expression of XIAP (inhibitor of apoptotic protein) and p53 phosphorylation were observed in the MUC1-positive transfectants in comparison with the MUC1-negative transfectants in response to suspension culture. Moreover, MUC1 cytoplasmic domain is known to regulate p53 activity either directly^22^ or indirectly^31^ and it has been reported that phosphorylation of p53 at different residues may have an impact on apoptosis.^32^ In the light of these reports, the increase in p53 phosphorylation in MUC1-positive cells and the substantial increase in expression of XIAP^33^ may, at least in part, provide an explanatory mechanism of the MUC1 cytoplasmic domain-associated anoikis resistance observed in this study. The marked accumulation of pro-caspase-3 observed in the MUC1-positive cells, which resist anoikis, in comparison with the MUC1-negative cells, which undergo anoikis, in response to suspension culture is very interesting. It indicates that one of the other possible mechanisms of the MUC1 cytoplasmic domain-mediated anoikis resistance may be associated with its inhibition of pro-caspase-3 proteolytic cleavage, hence caspase-3 activation.

Little was previously known of the influence of MUC1 on cellular anoikis. MUC1 transfection into ES-2 human ovarian tumour cells has been reported to decrease Annexin-V cell surface binding and increase chemoresistance of the cells to anticancer drugs in suspension.^34^ Transfection of MUC1 into rat 3Y1 fibroblasts was shown to increase the ability of the cells to grow in soft agar.^35^ During preparation of this manuscript, a study has reported that depletion of the MUC1 extracellular domain by transfection in human renal cells increases viability of the cells in response to culture under suspension condition, implying a role of the MUC1 extracellular domain in anoikis, but without further information regarding molecular mechanisms.^36^

The ability to grow under anchorage-independent conditions is one of the major hallmarks of transformed cells, key to this is the ability of the cancer cells to resist anoikis. Overexpression of MUC1 is a common feature in epithelial cancer cells. Overexpression of MUC1 has been shown to inhibit E-cadherin-mediated cell–cell interactions and to increase the ability of the cancer cells to detach from adjacent cells at primary tumour sites and to promote tumourigenesis.^15,37^ Interaction of cancer-associated MUC1 with circulating galectin-3, a galactoside-binding protein whose concentration is markedly increased up to 30-fold in the bloodstream of cancer patients,^21^ via expression of the oncofetal TF antigen on MUC1,^38^ induces MUC1 cell surface polarization and exposure of the cell surface adhesion molecules. This consequently results in increased homotypic aggregation and heterotypic adhesion of circulating tumour cells to the blood vascular endothelium and tumour cell spread.^19,20^ Thus, overexpression of MUC1 in epithelial cancer cells can influence several steps in tumourigenesis and metastasis and each of these is influenced not only by the MUC1 protein expression but also by the MUC1 localization/depolarization, its glycosylation patterns and the presence of its interacting proteins in the tumour microenvironment. For example, MUC1 overexpression prevents anoikis in suspended epithelial cancer cells as is shown here. But MUC1 overexpression also reduces adhesion of circulating tumour cells to the blood vascular endothelium, a metastatic step that is required for extravasation and establishment of a metastatic colony.^20^ On the other hand, interaction of MUC1 on circulating tumour cells with galectins induces MUC1 cell surface polarization to reveal the smaller cell surface adhesion molecules, causing increased cancer cell–endothelial adhesion^20,21^ and allowing increased formation of tumour emboli.^19^

Thus, expression of MUC1 prevents anoikis initiation of epithelial cells in response to loss of cell adhesion. The MUC1 extracellular domain makes a substantial contribution to this effect by maintaining the integrity and preventing activation of the cell surface anoikis-initiating molecules. This likely represents one of the mechanisms by which cancer cells avoid anoikis and may have important implications for the development of new therapeutic agents for cancer treatment.

## Materials and Methods

### Materials

The Caspase3/7 Glo kits, Caspase8 Glo kits and CellTiter-Glo Luminescent Cell Viability kit were obtained from Promega (Southampton, UK). Recombinant Fas-L was from PeproTech (London, UK). Antibodies against CD44 (BBA10), integrin*β*1 (MAB17782), E-cadherin (MAB1838), Fas (AF2267) and Fas-L (AF126) were from R&D Systems (Abingdon, UK). GenePOORTER-2 transfection reagent was from AMS Biotechnology (Abingdon, UK). FITC-Annexin-V/PI apoptosis detection kit was from Cambridge Biosciences (Cambridge, UK). The Calcein AM cell labelling solution was from Invitrogen (Paisley, UK) and the NECDS was from Sigma (Dorset, UK).

### Cells

Human colon cancer HCT116 and SW620 cells were obtained from European Collection of Cell Cultures (Salisbury, UK) and were cultured in McCoy's5a medium (HCT116) or Dulbecco's modified Eagle's medium (DMEM; SW620). MUC1 transfection of HBL-100 human breast epithelial cells and human melanoma A375 cells with full-length cDNA encoding MUC1 and the subsequent selection of the MUC1-positive transfectant HCA1.7+ (from HBL-100) and ACA19+ (from A375), and the negative revertant HCA1.7− (from HBL-100) and ACA19− (from A375) was described previously.^14^ The cell lines were last authenticated by DNA profiling (DNA Diagnostics Center, London, UK) in May 2014. MUC1 transfection of A375 cells with cDNA encoding only the MUC1 extracellular and transmembrane domains and subsequent selection of the MUC1-positive transfectant ATD2 was described previously.^39^

### Assessments of cell anoikis and viability

These assessments were conducted in cell suspension culture in poly-2-hydroxyethyl methacrylate (poly-HEMA)-coated plates. Briefly, 96- or 6-well plates were coated twice with 10 mg/ml poly-HEMA in 95% ethanol overnight. Cells were released by NECDS from the culture flasks, washed with PBS, resuspended to 5 × 10^5^ cells/ml with serum-free DMEM containing 0.5 mg/ml BSA and applied to the poly-HEMA-coated plates for various times at 37 °C. The cells were collected and the apoptotic (anoikis) cells were then measured either by FITC-Annexin-V/PI apoptosis detection kit with flow cytometry, or by the Caspase-Glo3/7 Assay kit according to the manufacturer's instructions. The viability of the cells was determined by the ATP detection kit (Invitrogen) as described in our previous study.^19^

### Analysis of the expression of cell surface adhesion molecules

The cells were released from the culture plates by either trypsin or NECDS and fixed immediately with 2% paraformaldehyde for 15 min at room temperature. After washing with PBS, the cells were incubated with 5% goat serum in PBS for 30 min. The cells were resuspended to 5 × 10^5^ cells/ml with 1% goat serum in PBS and incubated with antibodies (1 *μ*g/ml) against MUC1 extracellular repeat domain (B27.29), E-cadherin, CD44, integrin*β*_1_, Fas-L or control mouse IgG on a rotation platform for 1 h at room temperature. After three washes with PBS, FITC-conjugated secondary antibody (1 : 500 in 1% goat serum in PBS) was applied for 1 h. The cells were washed three times with PBS before flow cytometry analysis.

### Generation of full-length and tandem repeat-deleted MUC1 mutants

Generation of the MUC1 expression vectors for full-length MUC1 MUC1.Full and the extracellular domain-depleted MUC1 (MUC1FΔTR) was described previously.^40,41^ Each MUC1-expressing or control vector (1 *μ*g), pre-mixed with 25 *μ*l DNA Diluent and 5 *μ*l hydrated GenePOORTER-2 transfection reagent in 20 *μ*l serum-free medium for 10 min at room temperature, was added to 70–80% confluent HCT116 cells in 250 *μ*l antibiotics-free and serum-containing DMEM in 24-well plates for 24 h at 37 °C. The culture medium was replaced with serum-containing medium for 48 h before the cells were cultured in normal growth medium containing 600 *μ*g/ml G418 for 7–10 days at 37 °C. The cells were released and seeded at 30–50 cells/dish in 10 cm culture dishes in normal growth medium containing 600 *μ*g/ml G418. Single-cell clones were then selected with Cell Cloning Cylinders (Sigma), proliferated and analysed for MUC1 expression by immunoblotting with B27.29 (0.5 *μ*g/ml) and CT2 (0.25 *μ*g/ml) anti-MUC1 antibodies.

### Effect of exogenous Fas-L on caspase-8 activation under anoikis conditions

HCA1.7+ or HCA1.7− cells were released by NECDS and diluted into 2 × 10^5^/ml in serum-free DMEM. The cells were introduced (100 *μ*l/well) to poly-HEMA-pre-coated 96-well plates with or without introduction of 100 ng/ml Fas-L for 0 and 2 h followed by assessments of the cellular caspase-8 activity by Caspase-Glo 8 Assay kit.

### Protein array analysis of apoptosis-related proteins in cell response to anoikis culture

HCA1.7+/− cells were released by NECDS, washed and cultured at 1 × 10^5^ cells/ml in serum-free DMEM for 24 h in poly-HEMA-coated plates at 37 °C. The cells were collected and lysed with lysis buffer (provided by the Human Apoptosis Array Kit, R&D Systems) at 4 °C for 30 min. After centrifugation at 14 000 r.p.m. for 5 min, the supernatants were obtained and 500 *μ*g proteins from each sample were applied to the Human Apoptosis Array as described by the array kit. Each array contains 35 apoptosis-related proteins, each in duplicate (Bad, bax, Bcl-2, Bcl-x, pro-caspase-3, cleaved caspase-3, catalase, cIAP-1, xICAP-2, claspin, clusterin, cytochrome c, TRAIL R1/DR4, TRAIL R2/DR5, FADD, Fas/TNFSF6, HIF-1*α*, HO-1/HMOX1/HSP32, HO-2/HMOX2, HSP27, HSP60, HSP70, HTRA2/Omi, livin, PON2, p21/CDNK1A, phosphor-p53(S15), phosphor-p53(S46), phosphor-p53(S392), phosphor-Rad17(S635), AMAC/Diablo, surviving, TNF R1/TNFRSF1A and XIAP). The density of each apoptosis-related protein in the array was quantified by ChmiDoc XRS Imager (Bio-Rad, Hertfordshire, UK).

### Statistical analysis

Unpaired *t*-test for single comparison, one-way analysis of variance (ANOVA) followed by Newman and Keuls test for multiple comparisons (StatsDirect for Windows, StatsDirect Ltd; Sale, UK) were used where appropriate. Differences were considered significant when *P*<0.05.

## Abbreviations

CT: cytoplasmic domain
ECM: extracellular matrix
FADD: Fas-associated protein with death domain
NECDS: non-enzymatic cell dissociation solution
poly-HEMA: poly-2-hydroxyethyl methacrylate
TF: Thomsen–Friedenreich antigen (Gal*β*1,3GalNAc-*α*)
